# Anesthesia in Children with Neuroblastoma, Perioperative and Operative Management

**DOI:** 10.3390/children8050395

**Published:** 2021-05-14

**Authors:** Costanza Tognon, Rebecca Pulvirenti, Federica Fati, Federica De Corti, Elisabetta Viscardi, Andrea Volpe, Piergiorgio Gamba

**Affiliations:** 1 Anesthesiology Pediatric Unit, Women’s and Children’s Health Department, University Hospital of Padua, 35128 Padua, Italy; costanza.tognon@aopd.veneto.it; 2 Pediatric Surgery Unit, Women’s and Children’s Health Department, University Hospital of Padua, 35128 Padua, Italy; fedes.fati@gmail.com (F.F.); federica.decorti@aopd.veneto.it (F.D.C.); andrea.volpe@aopd.veneto.it (A.V.); piergiorgio.gamba@unipd.it (P.G.); 3 Hematology Oncology Division, Women’s and Children’s Health Department, University Hospital of Padua, 35128 Padua, Italy; elisabetta.viscardi@aopd.veneto.it

**Keywords:** neuroblastoma, anesthesia, post-operative pain, hypertension

## Abstract

Neuroblastoma (NB) is the most common extracranial, solid, pediatric malignancy and, despite the constant progress of treatment and development of innovative therapies, remains a complex, challenging disease causing major morbidity and mortality in children. There is significant variability in the management of neuroblastoma, partially due to the heterogeneity of the clinical and biological behavior, and partially secondary to the different approaches between treating institutions. Anesthesia takes an integral part in the multidisciplinary care of patients with NB, from diagnosis to surgery and pain control. This paper aims to review and discuss the critical steps of the perioperative and operative management of children undergoing surgery for neuroblastoma. Anesthesia and analgesia largely depend on tumor location, surgical approach, and extension of the surgical dissection. Attention should be paid to the physio-pathological changes on cardiovascular, gastrointestinal, and immune systems induced by the tumor or by chemotherapy. At the time of surgery meticulous patient preparation needs to be carried out to optimize intraoperative monitoring and minimize the risk of complications. The cross-sectional role of anesthesia in cancer care requires effective communication between all members of the multidisciplinary team.

## 1. Introduction

Neuroblastoma is the most common extracranial, solid, malignancy of childhood, accounting for 5 to 8% incidence and 15% mortality among pediatric malignancies [1]. Neuroblastoma is an embryonal tumor of the sympathetic nervous system and a heterogeneous disease, characterized by variable clinical and biological behavior. It can originate anywhere along the sympathetic chain such as a cervical, thoracic, retroperitoneal, or pelvic mass, affecting newborns, infants, or children; it can manifest as a small asymptomatic mass or as a large tumor with a major vascular encasement, spinal or bone marrow involvement. Prognosis ranges from spontaneous regression without treatment to aggressive, disseminated disease, refractory to multi-modal therapy including chemotherapy, surgery, and radiation. The result of this variability is a complex system of staging and pre-treatment risk stratification that tailors the intensity of treatment according to the patient’s stage, age and to the disease histology and biology [2].

Anesthesia takes an integral part into this articulated process, in a cross-sectional fashion, from diagnosis to the challenges of treatment, providing sedation and pain control, general anesthesia for surgery and invasive procedures, intensive and palliative care.

The aim of this paper is to review and discuss the critical points of the perioperative and operative management of neuroblastoma in infants and children.

## 2. Communication and Planning

Multidisciplinary team (MDT) case discussion is the first step for the preoperative planning of surgical procedures related to neuroblastoma, such as biopsy, primary mass resection, surgery of relapsed or metastatic disease. Members of the MDT are the oncologist, general and subspecialty surgeon, anesthesiologist, intensive care unit (ICU) specialist, radiologist, radiation oncologist, and nurse staff involved.

Clear communication between all the specialists is fundamental, discussion should cover patient, disease, and surgery-specific details. A documented plan should be available at the end of the MDT meeting giving clear information about patient preparation, surgery, anesthesia, and post-operative management.

Open communication to the patient’s family requires the discussion of the anesthesia plan, based on the expected procedure, patient’s age, and availability of venous access at the time of surgery. Possible risks and complications should be explained, as well as options for postoperative monitoring and pain management. The cultural or religious background of the family may need to be taken into account in this process.

## 3. Surgical Approaches and Anesthesia Considerations

### 3.1. Cervical Neuroblastoma

Primary cervical neuroblastoma covers approximately 3 to 5% of all locations, additional cases can be the result of metastatic spreading to neck lymph nodes [3]. The primary disease usually arises from the superior cervical ganglion of the sympathetic chain. The surgical approach is generally through a transverse cervical incision, the extension and position may change based on the size and depth of the mass or surgical expertise. The patient is usually positioned with the neck extended and the head turned on the opposite side of the mass. Critical steps of cervical NB resection are major vascular and nerve dissection, which can result in acute bleeding or nerve injury.

Airway compression or displacement is uncommon and rarely described in cervical NB major case series [4]. Depending on the tumor extension, an ultrasound-guided cervical block can be performed, that allows usually both deep and superficial anesthesia, granting good postoperative pain control in association with oral and intravenous analgesia.

### 3.2. Cervico-Thoracic Neuroblastoma

Primary cervicothoracic neuroblastoma usually arises from the stellate ganglion and progress extending in both directions, upward to the skull basis and downward to the posterior mediastinum. Access to the mass can be obtained through two main, although similar, approaches:

- the anterior cervical trans-manubrial, involving a lateral cervical incision extended through the midline of the sternum to the second intercostal space as a transverse thoracotomy [5];
- the “trap-door”, consisting of a supraclavicular incision extended through the midline of the sternum to the fourth intercostal space as a transverse thoracotomy [6].

In both techniques critical steps are the dissection of the vertebral arteries or brachial plexus roots, which again, can result in acute major bleeding in the chest or injuries of the brachial plexus roots or vagal nerve, determining pharyngeal dysfunction.

The first choice in cervicothoracic NB is general anesthesia associated with the insertion of an epidural catheter at the level of the thoracic spine, aiming to achieve analgesia both on the incision and on the site of chest tube insertion.

### 3.3. Thoracic Neuroblastoma

Primary thoracic neuroblastoma represents approximately 14% of possible locations [7]. It originates from the thoracic sympathetic ganglion chain, growing into the posterior mediastinum. Despite the close proximity, airway displacement has rarely been reported, when tumor-caused tracheal deviation or compression was present, the intraoperative course was still uneventful with no major interventions required. In case of airway involvement by the tumor, the use of a rigid bronchoscopy for intubation might be helpful [8].

The most common surgical approach is postero-lateral thoracotomy, which provides good exposure and control of the mediastinum and allows a safe tumor resection. Video-assisted thoracoscopic surgery is a valuable approach for smaller lesions and allows the patient to benefit from minimal incisions and a lower rate of postoperative pain [9]. Integral to the minimally invasive approach is One Lung Ventilation (OLV).

When thoracotomy is performed, anesthesia commonly involves general anesthetic drugs associated with the placement of an epidural catheter, as for the cervicothoracic tumors. General anesthesia associated with loco-regional blockade is generally performed during thoracoscopic procedures.

### 3.4. Abdominal and Pelvic Neuroblastoma

Retroperitoneum is the most common location of neuroblastomas, with adrenal glands being involved in 35% of cases [10]. Less than 5% of masses arise from the pelvic sympathetic ganglion. Surgical approaches change significantly according to the disease stage. Small tumors with no image-defined risk factors [11] can be faced with minimally invasive surgery, either laparoscopy or retroperitoneoscopy. Large masses with an extensive vascular encasement or lymph nodes dissemination require an open approach, consisting mainly of two different techniques: transverse abdominal laparotomy or thoracoabdominal exploration [12]. Isolated pelvic masses are generally approached through lower median laparotomy or laparoscopy.

A critical step for both open and minimally invasive approaches is vascular dissection. Acute bleeding is a frequent risk especially with extensive encasement of major retroperitoneal vessels such as the aorta, vena cava, and the renal hilum. Nephrectomy is a possible surgical complication as well as traumatic injury to the main branches of the abdominal aorta, like the celiac and the superior mesenteric artery.

Abdominal open surgery usually requires general anesthesia associated with a lumbar epidural catheter for intraoperative and postoperative analgesia. In case of a minimally invasive approach, general anesthesia with the addition of an ultrasound-guided loco-regional blockade is a feasible and reliable option.

## 4. Preoperative Assessment

Successful anesthesia in children with cancer requires a complete and thorough preoperative evaluation and neuroblastoma is no exemption. Full history and examination are necessary, special attention should be paid to the complications or comorbidities that can result from chemotherapy, radiation, surgery, or immunotherapy. As discussed previously, treatment of neuroblastoma has a variable structure reflecting the clinical variability of this condition. Treatment protocols may differ also according to the national or international study groups the treating institution belongs to.

For the aim of simplicity, we can state that neuroblastoma is a chemosensitive malignancy that generally responds to neoadjuvant chemotherapy and that can be broadly classified into high risk and non-high-risk disease. Non-high-risk tumors are amenable to upfront resection or benefit from low-intensity neoadjuvant chemotherapy. High-risk tumors require extensive neoadjuvant treatment. This is usually followed by local control of disease through surgery, eventually in association with radiotherapy. Intensification of neoadjuvant chemotherapy with the administration of additional cycles of therapy can be pursued if the response to the initial treatment is considered insufficient to achieve safe surgical local control. Adjuvant chemotherapy in association with bone marrow transplantation and immunotherapy follows, as consolidation and maintenance of treatment [13].

Children with neuroblastoma are expected to undergo surgery in two main moments of this treatment timeline: at presentation if a formal surgical biopsy is required for diagnosis or if a non-high-risk mass is considered feasible of upfront resection; at the end of neoadjuvant chemotherapy, usually after two to four months, for local control of the disease [2]. Further need for surgery can result in a case of relapse. Surgical complications requiring re-operation may develop unpredictably at any time, for example as a consequence of bleeding, infection, bowel or urinary obstruction, perforation, or fistula.

Basic preoperative workup includes full blood count with differential, complete biochemical profile including electrolytes, renal and liver function, coagulation profile. Tumor markers (urinary vanillomandelic and homovanillic acid, lactate dehydrogenase) are measured both at diagnosis and in preparation for surgery; serial measurements are performed after surgery and along with treatment as part of follow up. Cardiovascular function is assessed through a full set of vitals, ECG, and echocardiogram.

### 4.1. Imaging

Diagnostic and pre-operative imaging is reviewed at the time of MDT discussion. Children with neuroblastoma undergo cross-sectional imaging (CT scan, MRI scan) at the time of diagnosis to evaluate the primary tumor, local invasion, and metastatic disease. Metaiodobenzylguanidine scintigraphy (MIBG scan) is synergic to assess the presence of metastatic spread. PET scan has recently been introduced for metastasis evaluation as MIBG scan comes with a relatively low spatial resolution, resulting in limited sensitivity for small neoplastic lesions; NB specific radiotracers allow a better image resolution and a whole-body tomographic range.

Imaging is then repeated after neoadjuvant chemotherapy to establish a response to treatment. Urgent scans may be performed in the case of medical and surgical complications to confirm the diagnosis and to plan urgent procedures or surgery.

Performance of these imaging studies may require anesthesia assistance providing sedation to ensure the patient’s immobility and a good technical outcome of the scan. Sedation ensures also control of pain and anxiety. Anesthetic risk to sedation needs to be assessed and discussed in the case of acute illness or active severe complications.

A wide range of sedative drugs can be used in this setting, the choice mainly depends on the physician’s preference or expertise. The most commonly administered agents are propofol, ketamine, midazolam, fentanyl, remifentanil, and dexmedetomidine.

Sedation for diagnostic procedures is generally safe, rarely associated with major complications or serious adverse events, such as apnea, hypotension, laryngospasm, and bradycardia. Medical history, physical examination, current medical therapy, sedative to be used, type, and length of the procedure always need to be carefully evaluated to minimize procedure-associated risks [14].

Propofol is a short-acting agent that has proved to be safe, offering a shorter recovery time and a lower rate of minor complications compared to Ketamine and Midazolam. Short-acting time allows versatility, administration as a bolus or continuous infusion, allows tailoring the sedative effect to the duration of the diagnostic exam.

Hypertension has been described in children with cancer after sedation with ketamine during imaging studies [15], this has to be considered in patients with NB who may already be suffering from a tumor or catecholamine-related high blood pressure.

Patient sedation and contrast medium administration unavoidably require vascular access, which can be difficult to get in children with no central venous catheter available.

### 4.2. Chemotherapy Adverse Effect

Chemotherapy regimens for NB are modulated on the risk stratification. Main protocols for NB treatment indicate an association of cyclophosphamide, doxorubicin, carboplatin, and etoposide as the most effective choice for neoadjuvant or induction therapy.

Chemotherapy toxicity, especially for patients with high-risk disease, is responsible for a wide and complex spectrum of conditions, the effect of primary or metastatic disease often acts synergically affecting the bone marrow or cardio-vascular function.

Gastrointestinal toxicity is common among children undergoing chemotherapy for neuroblastoma, especially for those affected by high-risk tumors. Long-standing nausea, vomiting, diarrhea, and abdominal pain often lead to weight loss, malabsorption, failure to thrive, and cachexia. Profuse diarrhea is rarely consequent to tumor VIP hypersecretion. Severe metabolic alterations have to be detected in these cases and addressed timely in preparation for surgery.

Renal and hepatic toxicity can result from carboplatin and cyclophosphamide administration, respectively; chemotherapy-related impairment needs to be evaluated in the pre-operative work-up as it could affect the metabolism of some anesthetic agents. Renal damage could also be a result of tumor surgical dissection, especially in retroperitoneal NB surgery.

Doxorubicin has a well-recognized acute and long-term cardiac toxicity with a cumulative effect leading to cardiomyopathy and irreversible, congestive heart failure [16]. It should be avoided in the case of cardiomyopathy. Evaluation of cardiac function through echocardiographic left ventricle ejection fraction measurement is integral to the preoperative assessment.

High-risk NBs usually require a consolidation treatment comprehending myeloablative therapy associated to stem cell infusion; preferred myeloablative agents are busulfan and melphalan. Pulmonary toxicity represents one of the effects of this pharmacological association, ranging from mild pulmonary dysfunction to pulmonary fibrosis with restrictive lung disease. Respiratory impairment is more likely to occur during maintenance chemotherapy administration, nonetheless, it can be relevant when planning the post-operative diagnostic or therapeutic procedures requiring patient’s sedation.

Bone marrow toxicity related to myeloablative chemotherapy and bone marrow infiltration from metastatic disease can both result in anemia with low white cells or platelet count. Thrombocytopenia should be carefully considered as it may contraindicate major surgery and the placement of an epidural catheter. Leukopenia and neutropenia increase the risk of infection and impair the wound healing process.

### 4.3. Cardiomyopathy and Cardiac Toxicity

Cardiac function should be early and regularly monitored with an echocardiogram in neuroblastoma. Acute and chronic cardiac dysfunction can develop not only from doxorubicin toxicity but also from tumor-derived catecholamine hypersecretion.

A minority of patients affected by neuroblastoma show signs of reversible cardiomyopathy, structural myocardial changes develop due to high circulating levels of catecholamines [17]. Hyperadrenergic state generates chronic peripheral vasoconstriction, coronary spasm, chronic tachycardiomyopathy, beta-adrenergic receptors downregulation, oxidative stress, and calcium influx into sarcolemma in the cardiac muscle, leading to myocardial remodeling. Two clinical pictures have been described, left ventricular hypertrophy and hypertrophic-obstructive cardiomyopathy.

Neuroblastoma cardiomyopathy is generally found at diagnosis but it may develop during chemotherapy, no strict relationship with hypertension has been described. The condition is asymptomatic until a late stage and this remarks the importance of serial blood pressure monitoring and a complete cardiovascular evaluation for early diagnosis and safe administration of chemotherapy [18].

### 4.4. Hypertension

High blood pressure is uncommon in neuroblastoma, ranging from 10 to 19% of cases. Hypertension is secondary to two main mechanisms: renal vessel compression by a retroperitoneal mass or tumor secretion of catecholamines. The latter is considered to be rare in neuroblastoma, compared to other adrenal masses, because of the absence in neuroblastic cells of norepinephrine N-methyltransferase and intracellular granules for catecholamines synthesis and storage [19]. When present, catecholamine release peaks more frequently during neoadjuvant chemotherapy, due to tumor tissue remodeling, and at the time of surgery, when surgical manipulation of the mass takes place [18].

The anesthesiologist should always be prepared to manage sudden intraoperative cardiovascular fluctuations and perioperative assessment should be guided by a complete pre-operative work-up.

Blood pressure should be monitored throughout treatment to avoid cardiac, renal, and cerebral complications or end-organ disease. Pressure control facilitates anesthesiologic management reversing the hypovolemia due to chronic vasoconstriction and reducing intraoperative blood pressure fluctuation [20]. Catecholamine hypersecretion should be addressed with adequate preparation to optimize hemodynamic stability during surgery. Despite both the diagnostic work-up and treatment response monitoring, include measuring serum catecholamines levels (norepinephrine, epinephrine, dopamine) and their urinary metabolites (vanillylmandelic and homovanillic acid), this is rarely predictive of the risk of hypertension and its severity [18]. There are no established guidelines for the management of hypertension in patients with neuroblastoma, and small evidence supports a uniform approach, mostly related to the adult and to pheochromocytoma and adrenocortical tumors. Nonetheless, the benefits of effective blood pressure control have been documented in several case reports, for example in the reduction of perioperative mortality [19,21].

The most common medications reported to be effective in pressure control for catecholamine hypersecreting tumors are alpha1-adrenergic antagonists (phenoxybenzamine, phentolamine, prazosin, doxazosin) and beta-adrenergic blockers (atenolol, propranolol, labetalol).

Phenoxybenzamine in association with oral fluid intake increase has been described as a successful strategy in preparation for surgery, to both regulate blood pressure and achieve blood volume expansion, contrasting catecholamine-induced vasoconstriction and reducing the likelihood of post-resection hypotension. Beta-adrenergic blockers are added subsequently to control catecholamine and drug-related tachycardia [22]. Doxazosin has been used as an alternative to phenoxybenzamine with further benefits, it shows limited reflex tachycardia, reduced need for beta-adrenergic blockers, lower risk of arrhythmias, and shorter duration of action, limiting post-resection hypotension [20].

Intraoperative hypertension is rare, approximately 3–3.5% [22]. Hypertensive peaks are most likely to occur during anesthesia induction and tumor surgical manipulation. Administration of laryngeal local anesthesia—hypopharynx spray—helps in reducing the stress response from tracheal intubation. A wide range of short-acting drugs is available for intraoperative blood pressure control: alpha1-adrenergic antagonists (e.g., urapidil), beta-adrenergic blockers (e.g., esmolol), diuretics, calcium channel antagonists, sodium nitroprusside, phentolamine, adenosine, and fenoldopam [19,22].

## 5. Patient Preparation in the Operating Room

General anesthesia for NB surgery does not have any specific requirement compared to other interventions. Administration of a pre-anesthetic agent largely depends on centers’ protocols; the use of oral drugs, such as clonidine or midazolam, allows a quick and atraumatic intravenous induction.

Induction can be obtained either with intravenous or inhalational agents. The choice widely depends on the anesthesiologist’s expertise and personal preference. The advantage of intravenous induction is the rapid achievement of a deep level of anesthesia. Propofol has proved to be a good option because of its fast action, low incidence of intubation-induced bronchospasm, and strong antiemetic effect. Following induction, anesthesia is supplemented by intravenous opioids like fentanyl or remifentanil.

Pediatric tracheal intubation is a standardized procedure, for minimally invasive thoracic surgery one-lung ventilation is required to provide optimal exposure.

In high-risk resections, central venous access and additional stable peripheral cannulas are recommended for fluids and blood products infusion. A tunneled central venous catheter is already available in the vast majority of patients at the time of surgery as central venous access is positioned at diagnosis to proceed to chemotherapy. Continuous invasive arterial blood pressure monitoring and sampling are accomplished by the positioning of an arterial line, the radial artery is the first choice.

Preparation is completed by the insertion of a urinary catheter, temperature probe, and nasogastric tube when indicated. Especially for long procedures on lateral decubitus meticulous and extensive padding should be placed on pressure points to avoid pressure sores. Appropriate patient warming devices are mounted and stabilized without conflicting with exposure to the surgical field.

Detailed hemodynamic information may be useful in high-risk resections, in the event of compression or major bleeding from retroperitoneal vessels, and during manipulation of tumors secreting catecholamines. Continuative monitoring of the cardiac output and hemodynamic variations from beat-to-beat analysis of the arterial pressure profile is provided by invasive hemodynamic monitoring systems.

## 6. Postoperative Pain Management

A multimodal approach to pain control comprehends epidural or loco-regional analgesia associated with multi-agent intravenous medications: acetaminophen, non-steroidal anti-inflammatory drugs, and opioids. A combination of opioids and local anesthetics proved to be more effective for pain control and to be associated with a lower rate of adverse events compared to local anesthetics infusion alone [23].

### 6.1. Epidural Analgesia

The use of an epidural catheter in the postoperative management of children undergoing laparotomy or thoracotomy is well established [24]. However, epidural analgesia in the pediatric population has a unique risk profile, particularly, as a result of their different anatomy, younger children show decreased depth of the epidural space, the presence of a softer ligamentum flavum, and a longer angled spinous process with narrow interspaces. Ultrasound guidance allows one to overcome these technical challenges offering a good resolution of the central neuraxial anatomy and permits the limitation of the risk of damage of neural structures [25]. A recent report compares the management of postoperative pain control through epidural anesthesia and systemic opioids, showing a better result of pain control in children with epidural analgesia and reduced epidural complication when the catheter is placed by experienced providers [26].

Vital signs checks and focused neurological exams should be regularly performed. Major complications related to the use of an epidural catheter are dysfunction (15%) and dislodgement (20%). Occasionally, epidural catheters may lead to neurologic complications such as transient (0.1%) and permanent paralysis (0.006–0.02%) [24]. Contraindications to epidural analgesia are thrombocytopenia, heparin therapy, and intraspinal disease or surgery. Coagulopathy and bacteremia are relative contraindications, when present, risks and benefits need to be carefully considered [27].

Particular attention and close monitoring must be paid during epidural analgesia in patients with catecholamine secreting tumors; this analgesic approach has been described in one report as an independent risk factor for early postoperative hypotension, especially when pre-operative beta-blockers were needed [28].

### 6.2. Loco-Regional Analgesia

There is no wide evidence of the use of locoregional analgesia in the surgery of neuroblastoma. Nonetheless, the loco-regional blockade is an established approach to analgesia in endoscopic surgery and there is growing literature showing the effectiveness and safety of different peripheral nerve blocks in pediatric non-oncology surgery.

Minimally invasive procedures benefit from lower severity and shorter duration of postoperative pain. Analgesia combines surgical wound infiltration and loco-regional blockade with intravenous pain medication to achieve effective and well-tolerated pain control.

Regional blocks are safe and improve analgesia. Possible risks are needle trauma, compression/ischemia, and neurotoxicity. Compared to adult regional blocks, a lower concentration of local anesthetic is both sufficient to achieve analgesia and necessary to avoid toxicity. An appropriate weight-based dose has to be calculated for each patient as no standard is available for the pediatric population. Peculiar to children regional blocks is the rapid onset and the short duration of the anesthetic effect; commonly used agents are ropivacaine 0.2% or levobupivacaine 0.125 to 0.175% at roughly 0.3–0.5 mL kg^−1^. Clonidine and morphine can be used together to prolong the duration of analgesia [29].

Local anesthetic systemic toxicity presents with seizures, myocardial depression, and cardiac arrhythmias. It is more common in infants and special attention should be paid to this age group. Guidelines for treatment are available and include the administration of intralipid at the first suggestion of toxicity [27].

Contraindications to nerve block both in neonates and older children are similar and consist of absolute allergy to local anesthetic, guardian refusal, systemic infection, or infection at the insertion site.

Depending on the surgical site several loco-regional blockades are available.

The paravertebral blockade is commonly used for both thoracic and abdominal surgery as it provides good postoperative pain control and, compared to an epidural, is associated with fewer complications. The paravertebral blockade is unilateral and preserves the contralateral respiratory and sympathetic function [30].

In recent years, along with increasing use of ultrasound guidance, other approaches have been described, such as the Transverse Abdominis Plane (TAP), the Erector Spinae Plane (ESP), and the Quadratus Lumborum (QL) block. These approaches are a good option for procedures in non-high-risk neuroblastoma or when a limited surgical dissection is planned, for example, an open and minimally invasive biopsy of the primary tumor, minimally invasive upfront resection of low-risk masses, biopsy, or resection of metastatic lymph nodes. The TAP and QL blocks are suitable for retroperitoneal NB procedures, while the ESP block allows neck, thoracic and pelvic surgery to be covered.

TAP block is performed through the injection of local anesthetic between the internal oblique and the transversus abdominis muscle and it is usually adopted for analgesia of the lower thorax down to the L1-L2 level. Effectiveness is similar to epidural analgesia, with the benefits of being technically easier and resulting in a shorter hospital stay [31].

ESP block has been described in cervical, thoracic, abdominal, and limb surgery as a single-shot or continuous analgesia infusion. Advantages are the easy ultrasound-guided technique and a lower rate of complication, resulting from a safe distance between the target of the block and main vessels, pleura, and medulla. When paravertebral or epidural analgesia are contraindicated, the ESP block can be considered as a reliable alternative [32].

QL block has been firstly described as an alternative to TAP block for abdominal surgery. Quadratus lumborum muscle is a deeper target compared to transversus abdominis and its blockade seems to have a higher analgesic effect [33]. Also, positioning the patient in lateral decubitus results in an easier approach compared to the prone position required by the ESP block.

Cervical plexus block, either deep or superficial, can be used for surgeries involving the neck and the upper thoracic region.

Postoperative pain from thoracic, abdominal, or pelvic surgery can also be controlled through a subcutaneous analgesic system (SAS), in which one or more tunneled catheters are positioned along the surgical incision delivering a continuous infusion of local anesthetic, like ropivacaine. SAS showed better pain control compared to IV narcotics alone, with the benefits of avoiding the insertion of an epidural catheter and its related complications. In adults, the use of SAS has been associated with reduced administration of narcotics, contributed to early ambulation, and shorter hospital stay, with no difference compared to epidural in the incidence of analgesia-related adverse events. SAS could be a good option in patients with contraindications to epidural analgesias, such as coagulopathy, thrombocytopenia, and spinal disease. In children with cancer undergoing abdominal, pelvic, and thoracic procedures, the use of SAS proved to be equally effective and safer than epidural analgesia in post-operative pain control, showing a reduced incidence of pain device complications [24].

### 6.3. Intravenous Medications

Cancer-related pain is generally caused by direct nerve infiltration or tissue inflammation. Pain control is essential to ensure a good quality of life. Currently, a wide range of opioids is available including buprenorphine, codeine, fentanyl, hydromorphone, methadone, morphine, oxycodone, and tramadol. The most frequently used medications in the pediatric population are morphine and fentanyl [34].

Administration of morphine is considered to be safe in the pediatric population, side effects observed in neonates, infants, and children are similar to those observed in adults. Newborns do not seem to be more susceptible to respiratory depression than older children. The most common side effects of morphine are nausea and vomiting, sedation, pruritus, and urinary retention. Other possible side effects are constipation, broncho-constriction, respiratory depression, myoclonic movements, and physical and psychological dependence [35].

Recently, patient-controlled analgesia (PCA) has been gaining popularity, showing a high level of satisfaction with pain control both in children and parents.

Compared to epidural analgesia, PCA demonstrated comparable pain relief and cost-effect in children undergoing thoracotomy for cancer. In this scenario, the placement of an epidural catheter was associated with an increased cost of anesthesia, suggesting that PCA is a noninvasive and cost-effective alternative [25]. PCA emerged also as an excellent substitute to intravenous opioids in the postoperative pain management strategy, reducing the risk of respiratory depression [36].

## 7. Surgical and Anesthetic Side Effects

In recent years retrospective studies have been conducted, both in animal models and adult patients, with a focus on potential adverse effects of surgery, anesthetic drugs, and anesthetic techniques on tumor growth, progression of minimal residual disease, recurrence, and metastatic spread processes. The evidence is small and gives limited clarification of the underlying pathophysiology, more studies are needed to better understand the relationship of surgery and anesthesia with the immune response to cancer and tumor growth and progression.

The surgery itself can depress the innate immunity, increase the expression of pro-angiogenic factors and release growth factors promoting tumor progression. Alongside, anesthetic agents, such as halothane, isoflurane, and sevoflurane could impair the immune function. Furthermore, opioid analgesics could inhibit cellular and humoral immune function, while morphine may promote angiogenesis [37].

As a result of surgery- and anesthetic-derived immunosuppression, cancer cells released by surgical manipulation of tumor, may escape immune surveillance and induce metastasis [38].

In contrast to volatile agents, propofol seems not to depress NK-cells activity, have a potential role in reducing inflammatory cytokines, and at the same time, suppressing hypoxia-inducible factors activation. All these effects might lead to cancer spread inhibition [39].

Recent studies on animal models show a correlation of loco-regional anesthesia in cancer surgery with decreased incidence of metastatic recurrence from minimal residual disease and higher rates of relapse-free survival. This is described as the possible consequence of the reduction of surgical stress-response, lower opioid and/or volatile anesthetics requirements, and potential direct anti-cancer and anti-inflammatory effects of local anesthetic [40].

Regardless of these early results, further studies are necessary to define the underlying mechanisms related to tumor progression and metastatic spread. Current evidence does not justify any change in clinical practice.

## 8. Conclusions

Despite the constant progress and technological advances in the therapy of neuroblastoma, surgery and anesthesia remain milestones for the successful treatment of this challenging disease.

Knowledge of the critical points and structure of anesthesia for these complex procedures is helpful to all the health care professionals involved in cancer care and should be taken into consideration for the aim of improving quality of care, preventing morbidity and mortality, implementing communication, and patients’ quality of life.
